# Forsythiaside A improves Influenza A virus infection through TLR7 signaling pathway in the lungs of mice

**DOI:** 10.1186/s12906-022-03644-8

**Published:** 2022-06-22

**Authors:** Xiao Zheng, Ziqi Chen, Shanshan Shi, Huijun Yan, Junmei Zhou, Lifang Jiang, Hongli Wang, Guanghui Hou, Zhenyou Jiang

**Affiliations:** 1 Department of Microbiology and Immunology, College of Basic Medicine and Public Hygiene, Jinan University, Guangzhou, China; 2 Medical College, Sun Yat-Sen University, Guangzhou, China; 3 Key Laboratory of Tropical Disease Control (Sun Yat-Sen University), Ministry of Education, Guangzhou, China; 4 State Key Laboratory of Ophthalmology, Zhongshan Opthalmic Center, Sun Yat-Sen University, Guangzhou, 510632 China; 5 Zhuhai People’s Hospital (Zhuhai Hospital Affiliated with Jinan University), Zhuhai, China

**Keywords:** Influenza A virus, Forsythiaside A, TLR7^−/−^ mouse

## Abstract

**Background:**

Influenza A virus infection due to drug resistance and side effects of the conventional antiviral drugs yet remains a serious public health threat for humans and animals. Forsythiaside A is an effective ingredient isolated from the Chinese herbal medicine forsythia. It has various pharmacological effects and has a good therapeutic effect against a variety of infectious diseases. This study aimed to further explore the immunological mechanism of Forsythiaside A in the treatment of influenza virus-infected mice and its effect on the Toll-like receptor 7 (TLR7) signaling pathway in the lungs of these mice.

**Methods:**

C57/BL6J mice and TLR7^−/−^ mice were infected with the FM1 strains (H1N1 and A/FM/1/4) of the Influenza A virus. Each group of experimental mice were divided into the mock, virus, oseltamivir, and Forsythiaside A groups. Weight change, lung index change, and the mRNA and protein expression levels of key factors in the TLR7 signaling pathway were detected. Flow cytometry was used to detect the changes in the Th1/Th2 and Th17/Treg ratios.

**Results:**

After infection with the Influenza A virus, the weight loss of C57/BL6J mice treated with forsythoside A and oseltamivir decreased, and the pathological tissue sections showed that the inflammatory damage was reduced. The expression levels of the key factors, TLR7, myeloid differentiation factor 88(Myd88), and nuclear factor-kappa B (NF-κB) in the TLR7 signaling pathway were significantly reduced. Flow cytometry showed that Th1/Th2 and Th17/Treg ratios decreased after Forsythiaside A treatment. In the TLR7^−/−^ mice, there was no significant change after Forsythiaside A treatment in the virus group.

**Conclusions:**

Forsythiaside A affects the TLR7 signaling pathway in mouse lung immune cells and reduces the inflammatory response caused by the Influenza A virus FM1 strain in mouse lungs.

**Supplementary Information:**

The online version contains supplementary material available at 10.1186/s12906-022-03644-8.

## Background

**Fig. 1:**
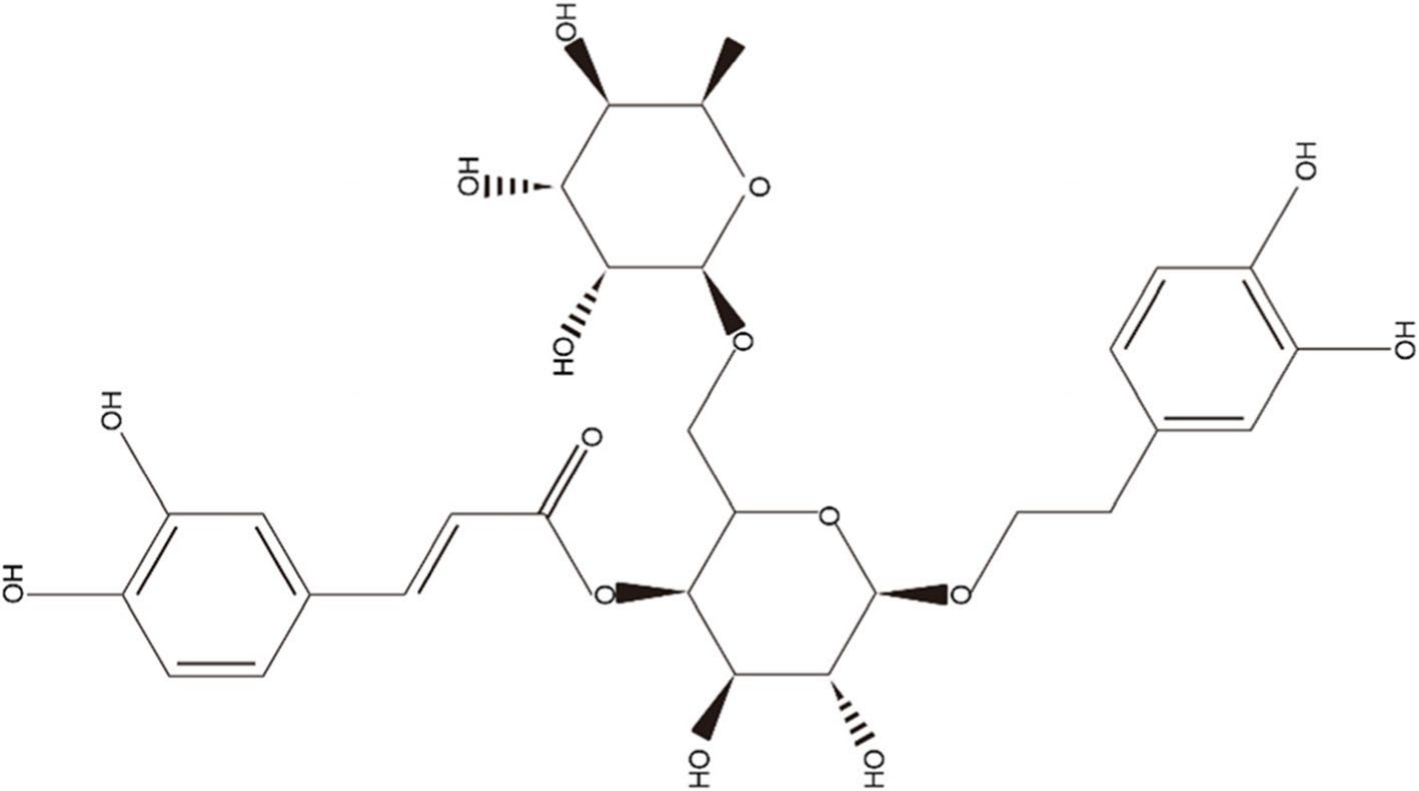
Chemical structure of Forsythiaside A

Forsythia, a well-known Chinese herbal medicine, belongs to the *Oleaceae* family and is widely used in China, Japan, and Korea. Owing to its antioxidant, antibacterial, and antiviral activities, forsythia plays an important role in the treatment of fever, inflammation, ulcers, and gonorrhea [1]. Up to now, more than three hundred components have been extracted from Forsythiae Fructus. Among these components, Forsythiaside A (Forsythoside A, C29H36O15) is the major bioactive index component of Forsythiae Fructus [2]. Forsythiaside A is based on two sugar groups and two aromatic rings, containing five alcohol acetoxy and four phenol acetoxy [3]. Its molecular structure is shown in Fig. 1. Forsythiaside A (FTA) is one of the main biologically active components of the plant and has in vivo and in vitro antibacterial, antioxidant, and antiviral activities [4, 5]. Forsythiaside A alleviates methotrexate-induced intestinal mucositis in rats by modulating the NLRP3 signaling pathways [6]. Forsythiaside A regulates the activation of hepatic stellate cells by inhibiting NOX4-dependent ROS [7]. In addition, Forsythoside A inhibits the avian infectious bronchitis virus in cell cultures [8].

The Influenza A virus is a common cause of seasonal epidemics [9]. Infected individuals exhibit mild symptoms, such as cough, sore throat, nasal discharge, fever, headache, and muscle pain. However, the symptoms can be more severe and lead to serious complications, such as bronchitis and pneumonia [10]. The global epidemic caused by human Influenza A viruses poses a serious threat to public health due to the lack of effective control methods. Currently, commonly used drugs, mainly including two types of antiviral drugs, (1) M2 ion channel inhibitors (amantadine and rimantadine) and (2) neuraminidase inhibitors (oseltamivir, zanamivir, peramivir, and laninamivir), have been approved for the treatment of influenza virus infections[10]. The efficacy of current treatment methods for influenza virus infections is reduced due to increased drug resistance. To combat the surge of virus outbreaks, new treatment methods are urgently needed. The influenza virus is a single negative-stranded RNA virus. When the virus infects the host, the pattern recognition receptors in host cells, including Toll-like receptors (TLRs) and the retinoic acid-inducing gene-I (RIG-I) [11], recognize the viral RNA and quickly initiate innate and adaptive immune responses. Toll-like receptor 7 (TLR7) recognizes single-stranded viral RNA and stimulates the downstream myeloid differentiation factor 88 (Myd88)-dependent signaling pathway. This ultimately leads to the secretion of cytokines and plays an important role in antiviral and antitumor processes [12]. The innate immune response is essential for controlling viral infections; however, excessive inflammation caused by a strong innate immune response is harmful to the host and is the cause of death in patients infected with Influenza A virus [13]. FTA has evident antiviral effects in vivo and in vitro [8, 14]. At present, the antiviral properties of Forsythiaside A mainly focus on the influenza virus, bovine viral diarrhea virus, avian infectious virus, and severe acute respiratory syndrome coronavirus 2 (SARS-CoV-2) [2]. However, its specific mechanism remains unclear. This study mainly assessed the effect of FTA on the TLR7 signaling pathway in the lung tissue of mice infected with the Influenza A virus.

## Materials and methods

### Virus

The virus infection experiments were performed in the Animal Biosafety Level 2 Laboratory (ABSL-2) of the Department of Microbiology and Immunology, Jinan University, Guangzhou, Guangdong Province. The virus was a mouse lung-adapted strain of the Influenza A virus FM1 strain (H1N1, A/FM1/1/4/ strain) provided by the Department of Microbiology and Immunology, School of Basic Medicine, Jinan University. The cryopreserved A/FM1/1/47 (H1N1) virus was revived, and chorionic intracellular allografts were inoculated into 10-day-old chicken embryos. After 48 h, the eggs were stored at 4 °C. Viruses were harvested under aseptic conditions and then aliquoted, titrated, and stored in liquid nitrogen.

### Drugs

Forsythiaside A standard product was purchased from the National Institute for the Control of Pharmaceutical and Biological Products (product batch number 111810- 201707). Oseltamivir phosphate (75 mg) was purchased from Yichang Dongyang Sunshine Pharmaceutical Co., Ltd. (product batch number H20065415).

### Virus titer

The mouse lungs were harvested on the seventh day after infection and homogenized in 1 ml Dulbecco’s Modified Eagle Medium. Plaque assay of Madin-Darby Canine Kidney (MDCK) cells was performed to determine the viral titer in the supernatant. The MDCK cell monolayer was seeded on a cell culture plate, and when the cells were overgrown, the lung tissue supernatant was serially diluted tenfold to obtain five concentrations of virus solution. Each well was washed with phosphate-buffered saline, and the diluted lung tissue homogenate was added. The cell culture plate was then incubated at 37 °C for 1 h. The mixture of 2% carboxymethyl cellulose and culture medium was covered, infected, and cultured for 4 days. Subsequently, the cells were fixed with 4% paraformaldehyde for 1 h. Sufficient crystal violet was added to the hole to cover the bottom; the cells were dyed for 15 min, then slowly rinsed with distilled water to remove the crystal violet solution, and the results were observed. The virus titer was calculated using the formula: PFU/ml = (number of plaques per well/virus inoculation volume per well) × virus dilution.

### Experimental animals and grouping

The experiment was conducted under the supervision and evaluation of the Experimental Animal Ethics Committee of Jinan University (IACUC-20191120-06). The C57BL/6 (wild-type [WT]) and TLR7^−/−^ mice were all healthy female mice aged 6–8 weeks and raised under specific pathogen-free conditions. The WT mice were purchased from the Guangdong Experimental Animal Center, and the TLR7^−/−^ mice were purchased from Taconic Farms in the United States. All knockout mice were backcrossed to the germ line of the WT mice for more than 10 generations. The mice were acclimated in a BSL2 container for at least 3 days prior to the experiment and housed in cages according to the strains. There were 24 WT and TLR7^−/−^ mice each, and they were randomly divided into four groups with six mice each, namely, the mock control, virus control, oseltamivir, and FTA groups.

### Experimental model establishment

Each group of experimental mice was labeled and weighed separately. The mock control, virus, oseltamivir, and Forsythiaside A groups were anesthetized and then infected with 50 μl of 5 × 10^3^ plaque-forming units (PFUs) of the virus through nasal drops. The mock control group was treated with an equal amount of saline. After the infection process was completed, the mice were returned to their independently ventilated isolation cages and kept normally. The mice were anesthetized with isoflurane. After introducing the virus, we observed and recorded the general living conditions, weight, and hair of the mice daily.

Antiviral drugs were started 24 h after nasal drip of the Influenza A virus FM1 strain. Each mouse in the mock control group and the virus control group was administered 0.4 ml/d of normal saline intragastrically. The oseltamivir group was administered a drug concentration of 30 mg/kg, 0.4 ml/d per mouse, and the Forsythiaside A group was administered a drug concentration of 20 mg/kg, 0.4 ml/d per mouse. The administration was continued for 5 days, and the general living conditions, body weight, and death of the mice were observed and recorded daily. On the sixth day after the drug treatment, the mice were euthanized by cervical dislocation after deep anesthesia, and tissues from the lung and spleen were collected.

### Body mass and lung index

The weight of the mice was recorded at the same time daily, and the weight change was recorded. The percentage of weight change was calculated using the following formula: percentage of weight change = (body weight on day N/body weight on day 0) × 100%. The dissected mouse lung tissue was washed twice with normal saline, the surface water was absorbed on a filter paper, and the lung weight was weighed and recorded. The lung index was calculated using the following formula: lung index = lung weight/body weight × 100%.

### Pathological tissue section

Fresh lung tissue samples were fixed with 4% paraformaldehyde, embedded in conventional paraffin, and sectioned to a thickness of approximately 5 μm. Two paraffin sections were randomly taken from tissue samples and stained with hematoxylin and eosin. The morphological changes in the lung tissues of each group of mice were observed and photographed under a light microscope.

### Quantitative reverse transcription polymerase chain reaction

Quantitative reverse transcription polymerase chain reaction (RT-qPCR) was used to detect the mRNA expression levels of TLR7, Myd88, and nuclear factor-kappa B (NF-κB) in each group and the relative amount of Influenza A virus replication in the lungs. After the homogenization of RNAiso Plus was added to the lung tissue, and total RNA was extracted. The purity and integrity of RNA were analyzed, and reverse transcription (Takara, Kusatsu, Japan) was performed. According to the manufacturer’s instructions, we subjected the obtained RNA to a genomic DNA removal reaction (42 °C for 2 min, 4 °C), followed by a reverse transcription reaction (37 °C for 15 min, 85 °C for 5 s, 4 °C). This was done using the reverse transcription product as a template and mouse glyceraldehyde 3-phosphate dehydrogenase (GADPH) as an internal reference. Primers for GADPH, TLR7, MyD88, NF-κB, and FM1 were designed and synthesized. The designed primers were used for PCR amplification to detect the primer amplification efficiency and product specificity. The primer sequences are shown in Table 1. The CFX Connect Real-Time PCR Detection System was used for real-time PCR detection, and each group was tested in three replicates. Finally, the cycle threshold (2^−ΔΔCt^) method was used to calculate the relative expression levels of the genes: △Ct = Ct target gene − Ct internal reference gene, and △△Ct = △Ct experimental group − △Ct control group. Each sample was measured three times.

**Table 1:** Primer sequence

Gene	Forward (5’-3’)	Reverse (5’-3’)
FM1	GACCAATCCTGTCACCTCTGAC	AGGGCATTNTGGACAAAGCGTCTA
TLR7	GGGTCCAAAGCCAATGTG	TGTTAGATTCTCCTTCGTGATG
Myd88	CGATTATCTACAGAGCAAGGAATG	ATAGTGATGAACCGCAGGATAC
NF-κB	ATTCTGACCTTGCCTATCTAC	TCCAGTCTCCGAGTGAAG
GADPH	CTGAGCAAGAGAGGCCCTATCC	CTCCCTAGGCCCCTCCTGTT

### Western blot detects the protein expression of key factors in the TLR7 signaling pathway

The mouse lung tissue was homogenized with radioimmunoprecipitation assay lysis buffer (LOT: 6,171,803, Multi Sciences, Hangzhou, China), and the total protein was extracted and quantified using a BCA protein quantification kit (LOT: A91041, Multi Sciences, Hangzhou, China). The proteins were separated by 10% sodium dodecyl sulfate–polyacrylamide gel electrophoresis and transferred to a polyvinylidene fluoride (PVDF) membrane. The PVDF membrane was blocked with 5% skimmed milk powder and combined with rabbit anti-mouse monoclonal antibodies TLR7 (1:1000, 2633S, CST, USA), MyD88 (1:1000, 4283S, CST, USA), NF-κB p65 (1:1000, 8242S, CST, USA), and GAPDH (1:1000, 2118S, CST, USA), and incubated overnight at 4 °C. Finally, the sections were incubated with goat anti-rabbit IgG that was labeled with horseradish peroxidase (LOT: A91053, Multi Sciences, Hangzhou, China). An electrochemiluminescence kit (LOT: A90844, Multi Sciences, Hangzhou, China) was used to detect protein bands, and the results were analyzed using the ImageJ software.

### Flow cytometry to detect the ratio of Th1/Th2 and Th17/Treg of T lymphocyte subsets in the mouse spleens

The mouse spleen tissue was collected, washed with serum-free 1640 medium, and homogenized. The supernatant was collected. The lymphocytes were separated with a lymphocyte separation solution (Multi Sciences, Hangzhou, China), and peripheral blood mononuclear cells were separated according to the manufacturer’s instructions. Moreover, the concentration was adjusted to 1 × 10^6^ cells/ml. Anti-CD4, CD25, FOXP3, IFN-γ, IL-4, and IL-17 antibodies (see Table 2 for antibody information) were used to detect different T cell subpopulations. Flow cytometry (Becton Dickinson Biosciences, Franklin Lake, NJ, USA) was used for detection, and the FlowJo V10 (FlowJo, Ashland, OR, USA) was used for the analysis.

**Table 2:** Antibodies used in flow cytometry

Antibody	Label	Company
CD4	PE	eBioscience
IL-17A	FTIC	eBioscience
CD25	PE-cyanine7	eBioscience
Foxp3	PE-cyanine5.5	eBioscience
IL-4	APC	eBioscience
IFN-gamma	APC	eBioscience

### Statistical analysis

Statistical analysis was performed using the GraphPad Prism software (version 8.0). The results were expressed as mean ± standard deviation. Multiple data sets were compared using one-way analysis of variance (ANOVA), and *P* < 0.05 was considered statistically significant.

## Results

### Weight change

**Fig. 2:**
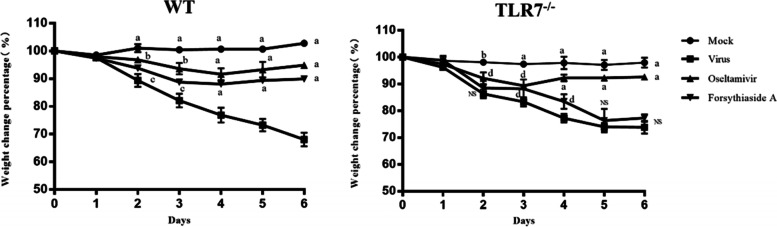
The effect of Forsythiaside A on the body weight of mice infected with influenza virus. Body weight change in WT and TLR7^−/−^ mice (*n* = 3). Statistical analysis was performed using repeated measures analysis of variance. Mock (mock control group), Virus (virus control group), Oseltamivir (oseltamivir group), Forsythiaside A (Forsythiaside A group). a: ****P < 0.0001, b: ***P < 0.001, c: **P < 0.01, d: *P < 0.05, NS = not significant, *P* > 0.05

After the influenza virus infection, the survival and body weight of the mice were monitored daily (Fig. 2). The virus-infected group of WT and TLR7^−/−^ mice showed weakened activity and delayed response on the second day after virus infection, whereas the mock group did not demonstrate this change. After the third day, the virus group of WT and TLR7^−/−^ mice showed fluffy hair, loss of appetite, and rapid weight loss. The body weight of mice in the mock group increased slightly or remained unchanged. The oseltamivir and FTA groups of WT mice demonstrated significant improvements in symptoms and reduction in weight loss. This difference was significant as compared to the virus group (*P* < 0.05). However, there was no significant difference between the virus and FTA groups of TLR7^−/−^ mice (*P* > 0.05).

### Histopathological analysis of the lung tissue and lung index

**Fig. 3:**
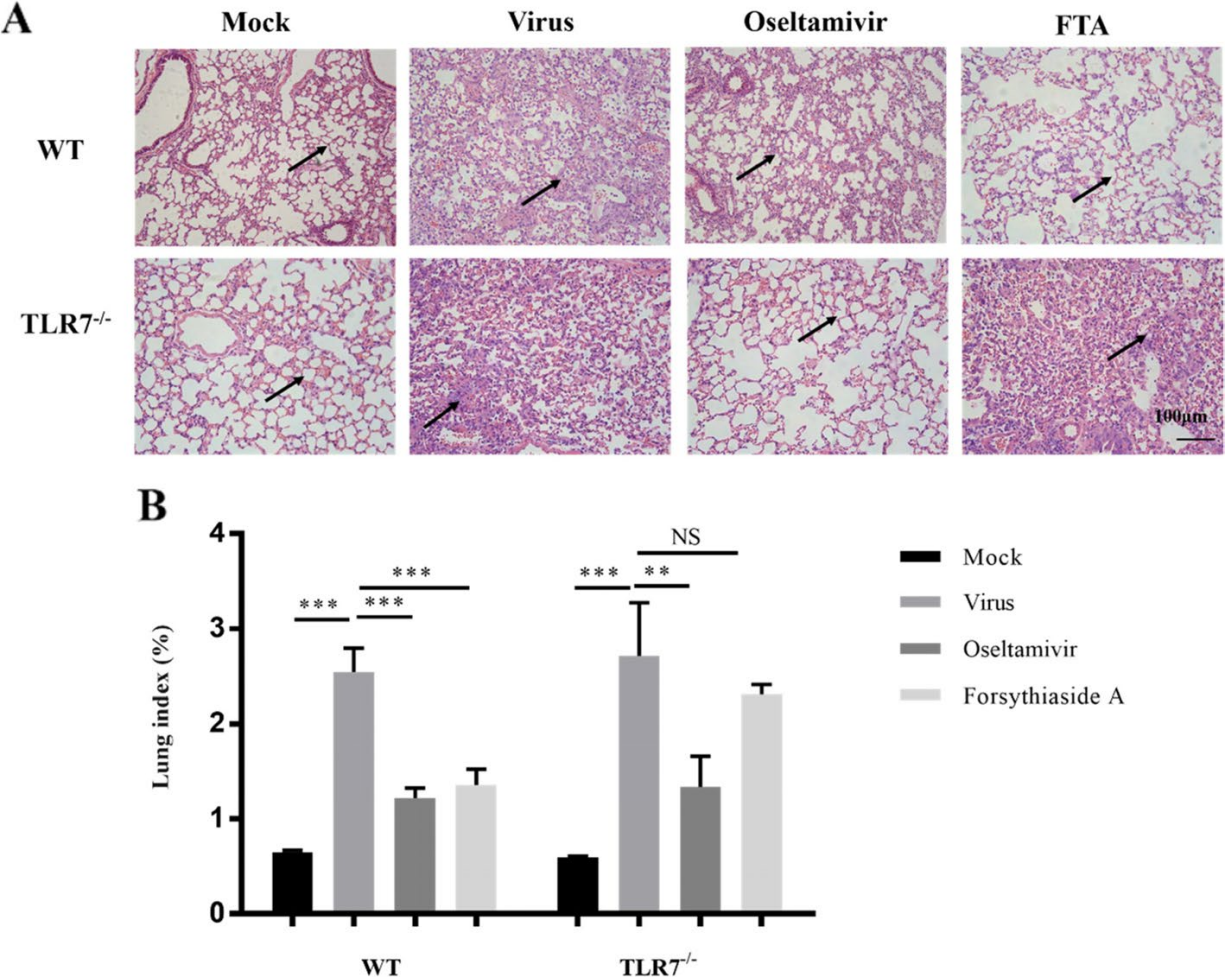
Lung pathological changes after influenza virus infection in mice. **A** Pathological changes of lung tissue in WT mice and TLR7.^−/−^ mice. On the 6th day after infection, lung tissue sections were collected for histopathological analysis (*n* = 6), Bar = 100 μm. **B** The effect of forsythiaside A on the lung index of mice infected with influenza virus. Lung index = lung weight/body weight × 100% (*n* = 3). *P < 0.05, **P < 0.01, ***P < 0.001, NS = not significant, *P* > 0.05

The lung tissue of healthy mice has a complete alveolar structure, regular morphology, complete bronchial mucosal epithelium and mucosal muscularis, and no surrounding inflammatory cell infiltration. After the mice were infected with the influenza virus, the lung pathological sections showed diffuse damage in the alveoli, alveolar diaphragm, and bronchi. Dense lymphocytic infiltration of the lung interstitium was observed. The pathological sections of the mock groups of WT and TLR7^−/−^ mice showed normal lung tissue. In contrast, the virus groups showed diffuse injury in the alveoli, alveolar septum, and bronchus, and dense lymphocytic infiltration of the lung stroma. The histopathological sections of the mock group of WT and TLR7^−/−^ mice showed normal lung tissue. In contrast, the virus group showed diffuse damage in the alveoli, alveolar diaphragm, and bronchus, and dense lymphocytic infiltration of the lung interstitium. Inflammatory cell infiltration in the lung tissues of the oseltamivir and FTA groups of WT mice was significantly reduced, and the bronchial mucosa was relatively intact. In the FTA group of TLR7^−/−^ mice, the blood vessels were dilated and congested, the alveoli were dilated or even broken, and pulmonary interstitial edema and inflammatory cell infiltration in the alveoli were evident. In summary, FTA can significantly reduce lung inflammation in WT mice, but cannot reduce lung inflammation in TLR7^−/−^ mice (Fig. 3A). The lung index can be used to describe the severity of lung inflammation. The greater the lung index, the more severe the lung inflammation. Compared with the virus group, the lung index of the oseltamivir and FTA groups was significantly lower in the WT mice. There was no significant difference between the lung index of the FTA and virus groups of TLR7^−/−^ mice (*P* > 0.05). The results showed that FTA significantly reduced the lung index of WT mice, but not of TLR7^−/−^ mice (Fig. 3B).

### Replication of the Influenza A virus in the mouse lungs

**Fig. 4:**
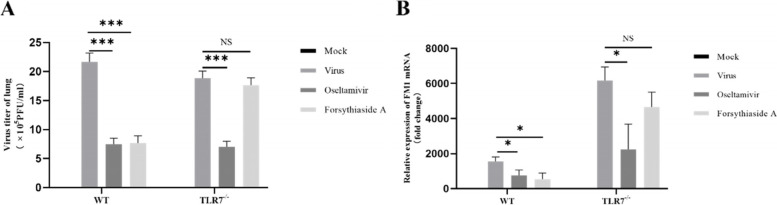
Influenza FM1 viral load in lung tissue. **A** Shows the viral titer in the lung tissue of WT mice and TLR7^−/−^ mice (*n* = 3). **B** Shows the FM1 mRNA expression level of influenza virus in the lung tissue of WT mice and TLR7.^−/−^ mice (*n* = 3). *P < 0.05, **P < 0.01, ***P < 0.001, NS = not significant, *P* > 0.05

The plaque formation experiment showed replication of the virus in mice (Fig. 4A). At the same time, RT-qPCR was used to detect FM1 mRNA expression in the mouse lungs (Fig. 4B). Compared with the virus group, the expression of FM1 mRNA and virus titer in the oseltamivir group decreased in the WT and TLR7^−/−^ mice. The expression of the virus in the FTA group of WT mice was significantly reduced, whereas the expression of FM1 virus in TLR7^−/−^ mice was not significantly different from that in the virus group. These results indicate that FTA has an effect on virus expression in mice infected with the Influenza A virus and affects virus replication through the TLR7 signaling pathway.

### Relative mRNA and protein expression of key factors in the TLR7 signaling pathway

**Fig. 5:**
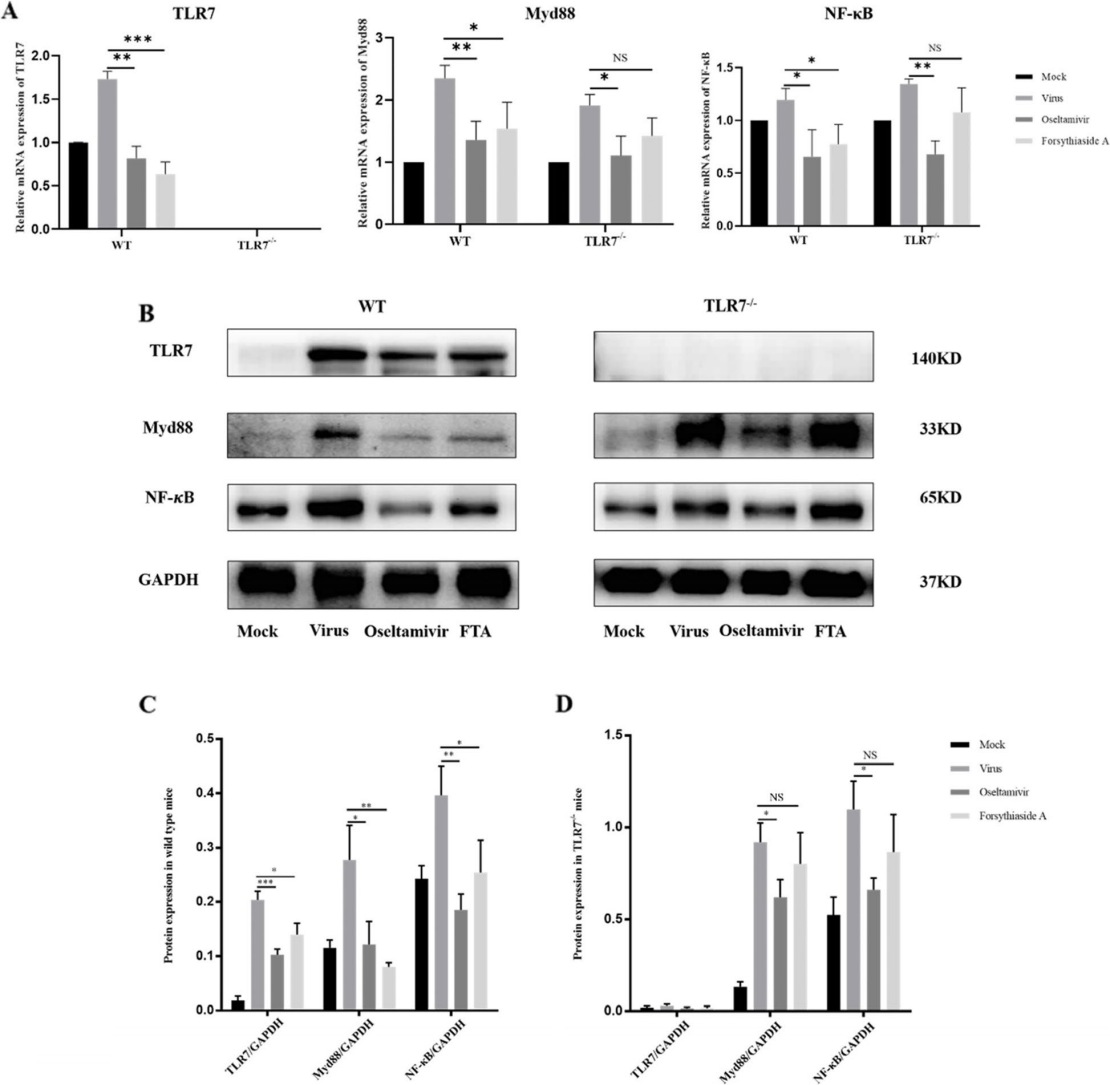
Relative mRNA and protein expression of key factors in the TLR7 signaling pathway. **A** Represent the relative expression of TLR7, Myd88 and NF-κB mRNA in the TLR7 signaling pathway in WT mice and TLR7^−/−^ mice (*n* = 3); **B** shows the expression of TLR7, Myd88, and NF-κB proteins in WT mice and TLR7^−/−^ mice; **C** and **D** show statistical graphs of TLR7, Myd88, and NF-κB protein expression in WT mice and TLR7.^−/−^ mice (*n* = 3). *P < 0.05, **P < 0.01, ***P < 0.001, NS = not significant, *P* > 0.05

The expression levels of TLR7, Myd88, and NF-κB p65 mRNA were determined to study the effect of FTA on the TLR7 signaling pathway of viral infection (Fig. 5A). In the virus group of WT and TLR7^−/−^ mice, the relative expression levels of TLR7, Myd88 and NF-κB p65 mRNA were higher, and the difference was statistically significant. In the WT mice, the levels in the oseltamivir and FTA groups decreased significantly. However, in the TLR7^−/−^ mice, the relative expression levels of Myd88 and NF-κB p65 mRNA in the oseltamivir group decreased significantly, and there was no significant difference in the FTA group. The statistical results showed that FTA decreased the gene expression level of the TLR7 signaling pathway to varying degrees as compared with oseltamivir. We detected the expression levels of TLR7, Myd88, and NF-κB p65 proteins in the TLR7 signaling pathway in the lung tissues (Fig. 5B). In the lung tissues of the WT mice, the expression levels of TLR7, Myd88, and NF-κB p65 proteins in the oseltamivir and FTA groups were significantly lower than those in the virus group (Fig. 5C). In the TLR7^−/−^ mice, the expression levels of Myd88 and NF-κB p65 protein in the oseltamivir group were lower than those in the virus group. However, the FTA group demonstrated no significant difference from the virus group (Fig. 5D). The results showed that FTA downregulated the protein expression levels of genes related to the TLR7 signaling pathway.

### The expression of Th1/Th2 and Th17/Treg in spleen cells

**Fig. 6:**
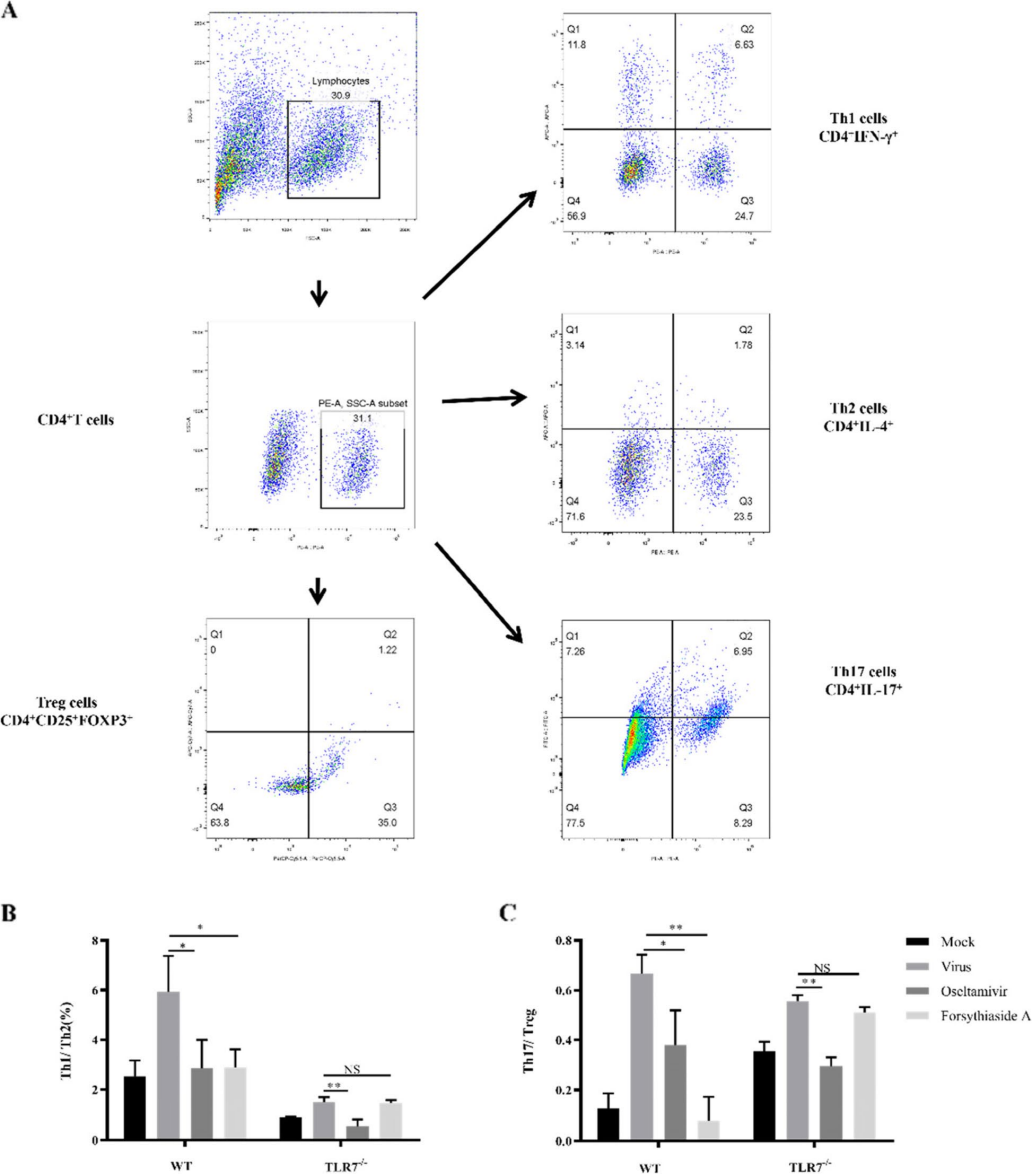
The ratio of Th1/Th2 and Th17/Treg in spleen tissue. **A** Represents flow cytometry analysis of Th1, Th2, Th17, and Treg cell subsets; **B** and **C** represent the ratio of Th1/Th2 and Th17/Treg cell in WT mice and TLR7-/- mice, respectively (*n* = 3). *P < 0.05, **P < 0.01, ***P < 0.001, NS = not significant, *P* > 0.05

To verify the inflammatory response in mice, we used flow cytometry to detect the expressions of Th1, Th2, Th17, and Treg cells obtained from the spleen tissue of the mice (Fig. 6A). The balance between the Th1/Th2 and Th17/Treg cells reflected the stability of immune function. Compared with the virus group, the Th1/Th2 and Th17/Treg ratios of the oseltamivir and FTA groups of WT mice were significantly lower. In the TLR7^−/−^ mice, the Th1/Th2 and Th17/Treg ratios in the oseltamivir group were significantly reduced, whereas the ratios in the FTA group were not significantly different from those in the virus group (Fig. 6B and C).

## Discussion

The Influenza A virus is the main pathogen of respiratory tract infections, causing widespread prevalence of acute respiratory infectious diseases in humans and animals worldwide [15]. It has become one of the most variable pathogens threatening human health and leads to serious diseases and deaths almost annually [16], which is a huge burden on the global healthcare system. The influenza virus is mainly transmitted through respiratory tract by droplets. Once the influenza virus infects respiratory epithelial cells, it multiplies and produces a large number of virus particles within a few hours. The infectious particles are released from the apical plasma membrane of epithelial cells into the airway by budding. The virus spreads rapidly in the lungs due to rapid infection of adjacent cells. Influenza A or B virus infection usually manifests as an acute self-limiting upper respiratory tract disease, characterized by fever, cough, sore throat, and physical discomfort [17].

Traditional Chinese medicine plays an important role in the treatment of various diseases. FTA is the main active ingredient isolated from Forsythia and modern pharmacological studies have confirmed that FTA exhibits superior activity in the treatment of various diseases, including inflammation, viral infection, neurodegeneration, oxidative stress, liver damage and bacterial infection [2]. Furthermore, several studies have confirmed that Forsythiaside A exhibits various pharmacological properties by regulating multiple signaling transduction pathways, such as NF-κB, MAPK, JAK/STAT, Nrf2, RLRs, TRAF, TLR7, and ER stress [14, 18–21].

Frequent mutations in influenza viruses lead to ineffective vaccines, while antiviral drugs develop resistance and adverse reactions. Traditional Chinese medicine monomer FTA has significant antiviral effect in vitro and in vivo experiments. At the same time, in vivo experiments have verified that it has an antiviral effect on the influenza virus [22]. With increasing number of reports on the effects of FTA on the immune function of the body (including non-specific immunity, specific immunity, and secretion of cytokines), research on FTA-related topic has gradually increased. In this experiment, WT mice were infected with influenza virus FM1 strain and treated with FTA to verify the therapeutic effect of FTA on influenza virus infection and its influence on the immune mechanism. In addition, TLR7^−/−^ mice were also used to demonstrate the effect of FTA on the TLR7 signaling pathway in the lungs of mice infected with the FM1 strain of Influenza A virus. Meanwhile, the neuraminidase inhibitor oseltamivir, a mainstream drug for influenza, was used as a positive control [23].

First of all, in order to determine whether the influenza A virus model can be successfully established in WT mice after infection with influenza A virus, we detected the virus titer in the lungs of mice by the plaque formation experiment. We also detected the mRNA expression level of the virus by RT-qPCR. The mRNA expression in the virus group was significantly higher than that in the control group. WT mice administered FTA and oseltamivir did not show statistically significant differences in terms of body weight and lung index. However, the pathological results showed that the alveolar structure and degree of inflammation in the FTA group mice were relieved. Thus, FTA can control the inflammatory response caused by Influenza A virus infection.

When the influenza virus infects the body, the non-specific immune response is the first to get activated and play a role in the initial stage of infection, which can control the viral replication and disease spread [24]. The expression of TLR7 pathway signal-related factors increases during influenza virus infection [25]. TLR7 is a member of the TLR family and acts as a natural barrier against single-stranded RNA viruses by activating the downstream signal protein of the downstream adaptor protein Myd88 [11]. Myd88 recruits and phosphorylates interleukin receptor-associated kinase and then combines with TNFR-associated factor to activate the NF-κB signaling pathway. This leads to the production of pro-inflammatory cytokines and chemokines and induces innate and adaptive immune responses. This signaling pathway plays an important role in the antiviral and antitumor processes [26]. The innate immune response is crucial for the control of viral infections. Influenza virus infection leads to an increased production of cytokines and chemokines, resulting in a “cytokine storm” that is thought to be associated with pathogenesis and poor prognosis [13]. Morever, it is detrimental to the host and is one of the leading causes of death in patients infected with influenza A virus [27].

In WT mice infected with FM1 influenza strain, FTA may play an anti-influenza effect by downregulating key factors such as TLR7, Myd88 and NF-κB in the TLR7 signaling pathway. To verify that FTA treatment produces efficacy through TLR7 signaling in lung immune cells, we infected TLR7-/- mice with the FM1 influenza strain and treated them with FTA. In the TLR7^−/−^ mice, the experimental results showed that the FTA treatment had no therapeutic effect and there was no significant statistical difference between the expression of related factors in the TLR7 signaling pathway in the FTA and virus groups. This indicates that FTA plays a role by affecting the expression of related factors in the TLR7 signaling pathway.

Different subsets of T helper cells express different cytokines and transcription factors and respond to different types of pathogens. Their unique differentiation modes are determined by transcription factors and genes transcribed by the transcription factors. According to phenotypic and functional characteristics, CD4^+^ T cells can be a subset of Th1, Th2, Th17, or Treg cells. The balance of these four immune cells plays an important role in hypersensitivity and autoimmune diseases [28]. Under normal conditions, the Th1/Th2 ratio is relatively stable and in a state of dynamic equilibrium. An imbalance in this ratio often causes immune imbalance, leading to the occurrence of various diseases [29]. Th1 cells expressing IFN-γ help to eliminate intracellular pathogens, and Th2 cells that affect antibody class switching express high levels of IL-4. Th17 cells produce IL-17A, a typical inflammatory factor. CD4^+^ FOXP3^+^ Treg cells are immunosuppressive cells that play an important role in maintaining immunological self-tolerance and controlling immune responses to induce tissue damage and can suppress immune reactions. The differentiation of Treg and Th17 cells inhibits each other and is negatively regulated. Under normal circumstances, initial CD4^+^ T cells differentiate into Treg cells. In the presence of infection or inflammation, the initial CD4^+^ T cells differentiate into Th17 cells, thereby inducing a chronic inflammatory response. Therefore, Th17/Treg cells represent an indicator that regulates the balance of inflammation [30]. The Th1/Th2 and Th17/Treg ratios were detected by flow cytometry, and it was found that the Th1/Th2 and Th17/Treg values in the virus control group were higher as compared to the mock control group. This indicates that infection with the Influenza A virus can promote CD4^+^ T cell differentiation toward Th1 and Th17 cells, and enhance the body’s pro-inflammatory response. After treatment with FTA in the WT mice, the ratio of Th1/Th2 and Th17/Treg in the FTA group was significantly lower than that in the virus group, and the difference was statistically significant (*P* < 0.05). After FTA treatment in the TLR7^−/−^ mice, the ratios were still higher than those in the control group (*P* > 0.05).

Forsythiaside A is the component at the highest level in Forsythia suspensa, which has been applied as one of the marker components to evaluate the quality of Forsythia suspensa and preparations containing Forsythia suspensa [31]. FTA is a phenylethanoid glycosides, they are glycosides with phenylethyl alcohol and glycosyl moieties. The presence of phenolic hydroxyl groups enhances the antioxidant activity of the compounds. The skeleton of phenylethanoid glycosides includes three parts: phenylethyl alcohol, caffeic acid and a glycosyl group. According to the number and type of glycosyl groups on the mother nucleus, phenylethanoid glycosides can be divided into phenylethanol monoglycosides, disaccharides, trisaccharides and tetraglycosides. FTA is a disaccharide including α-L-Rha with various pharmacological activities. Due to the small size, simple structure, and strong variability of the virus, the development of antiviral drugs faces substantial challenges [32]. In this study, we found that FTA improves influenza A virus infection through TLR7 signaling pathway in the lungs of mice. Therefore, FTA may be a potential influenza virus treatment drug.

## Conclusions

The experimental results indicate that FTA can enhance the immune regulation function of influenza virus-infected mice by downregulating the TLR7 signaling pathway, reducing the inflammatory response, and reducing the immunopathological damage in the virus-infected lung. This shows the potential of the traditional Chinese medicine monomer FTA to treat influenza and lays a firm foundation for drug research and development. However, the specific application of this monomer requires further study.

## Supplementary Information

**Additional file 1: Supplementary Figure 1.** This figure represents the original picture of GAPDH in the WT mice of Fig. 5A in the manuscript. **Supplementary Figure 2.** This figure represents the original picture of TLR7 in the WT mice of Fig. 5A in the manuscript. **Supplementary Figure 3.** This figure represents the original picture of Myd88 in the WT mice of Fig. 5A in the manuscript. **Supplementary Figure 4.** This figure represents the original picture of NF-κB in the WT mice of Fig. 5A in the manuscript. **Supplementary Figure 5.** This figure represents the original picture of GAPDH in the TLR7^−/−^ mice of Fig. 5A in the manuscript. **Supplementary Figure 6.** This figure represents the original picture of TLR7 in the TLR7^−/−^ mice of Fig. 5A in the manuscript. **Supplementary Figure 7.** This figure represents the original picture of Myd88 in the TLR7^−/−^ mice of Fig. 5A in the manuscript. **Supplementary Figure 8.** This figure represents the original picture of NF-κB in the TLR7^−/−^ mice of Fig. 5A in the manuscript.

**Additional file 2.** Raw data RT-PCR.

## Data Availability

The data used to support the findings of this study are available from the corresponding author upon request.

## Abbreviations

TLR7: Toll-like receptor 7
Myd88: Myeloid differentiation factor 88
NF-κB: Nuclear factor-kappa B
FTA: Forsythiaside A
TLRs: Toll-like receptors
RIG-I: Retinoic acid-inducing gene-I
WT: Wild-type
MDCK: Madin-Darby Canine Kidney
PFU: Plaque-forming units
RT-qPCR: Quantitative reverse transcription polymerase chain reaction
GADPH: Glyceraldehyde 3-phosphate dehydrogenase
PVDF: Polyvinylidene fluoride
