# Supplementary material for: Forsythiaside A improves Influenza A virus infection through TLR7 signaling pathway in the lungs of mice
Source: BMC Complement Med Ther. 2022 Jun 22;22:164. doi: 10.1186/s12906-022-03644-8 (PMC9214192; doi:10.1186/s12906-022-03644-8)
Supplement: Supplementary file 1 — Additional file 1: Supplementary Figure 1. This figure represents the original picture of GAPDH in the WT mice of Fig. 5A in the manuscript. Supplementary Figure 2. This figure represents the original picture of TLR7 in the WT mice of Fig. 5A in the manuscript. Supplementary Figure 3. This figure represents the original picture of Myd88 in the WT mice of Fig. 5A in the manuscript. Supplementary Figure 4. This figure represents the original picture of NF-κB in the WT mice of Fig. 5A in the manuscript. Supplementary Figure 5. This figure represents the original picture of GAPDH in the TLR7−/− mice of Fig. 5A in the manuscript. Supplementary Figure 6. This figure represents the original picture of TLR7 in the TLR7−/− mice of Fig. 5A in the manuscript. Supplementary Figure 7. This figure represents the original picture of Myd88 in the TLR7−/− mice of Fig. 5A in the manuscript. Supplementary Figure 8. This figure represents the original picture of NF-κB in the TLR7−/− mice of Fig. 5A in the manuscript. [file 12906_2022_3644_MOESM1_ESM.pdf]

Additional file

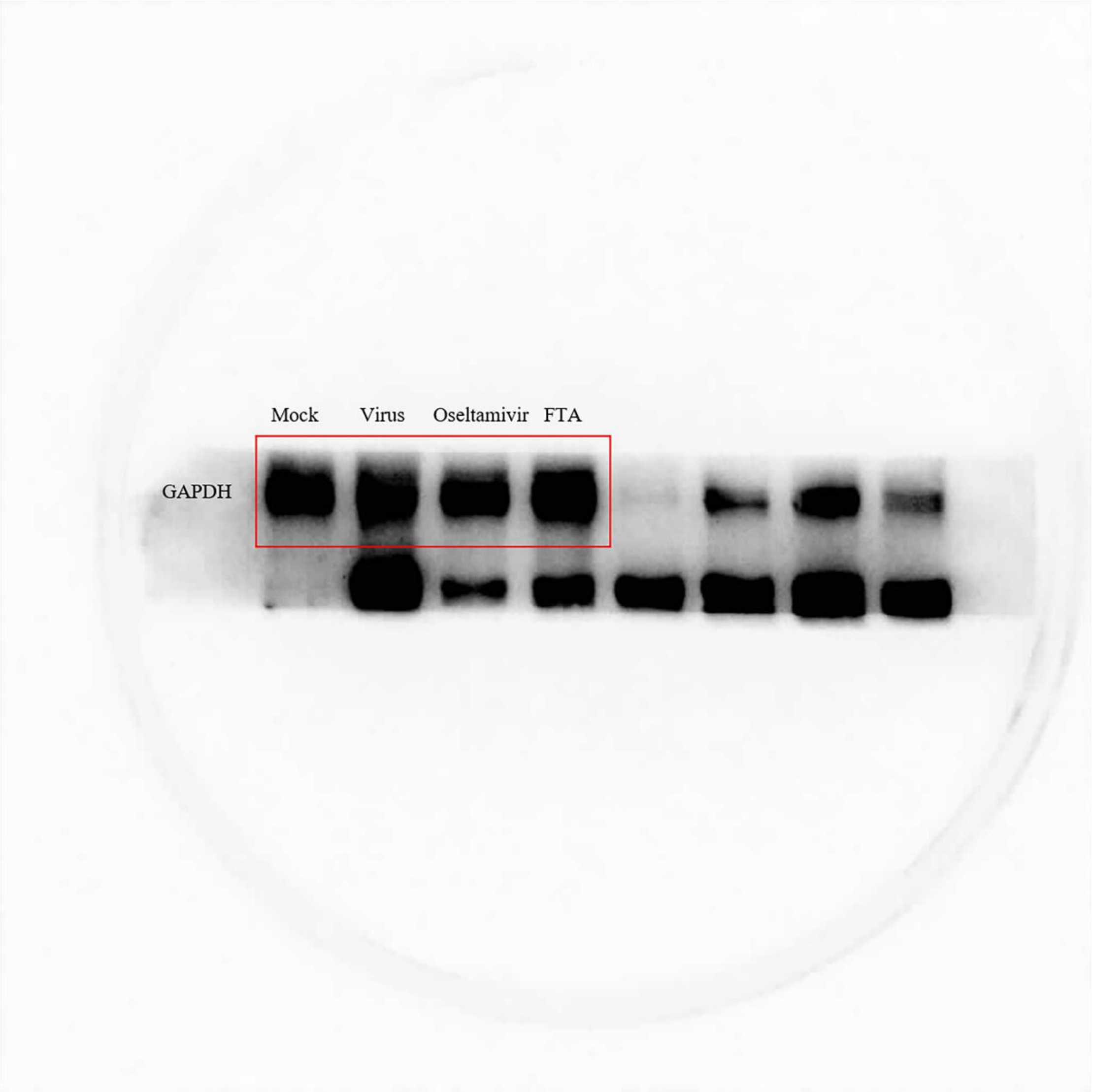

Supplementary Figure1. This figure represents the original picture of GAPDH in the WT mice of Figure 5A in the manuscript.

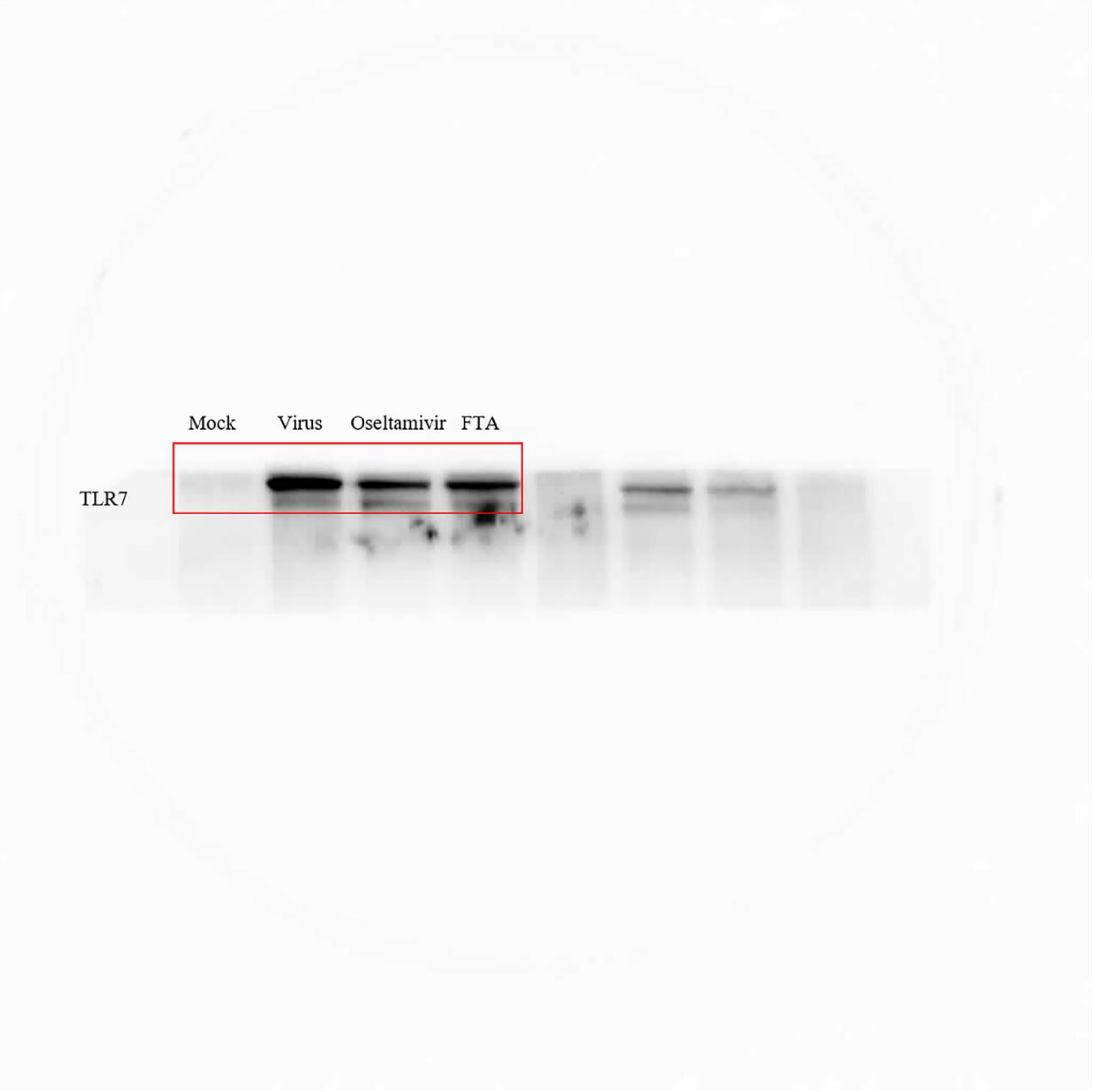

Supplementary Figure2. This figure represents the original picture of TLR7 in the WT mice of Figure 5A in the manuscript.

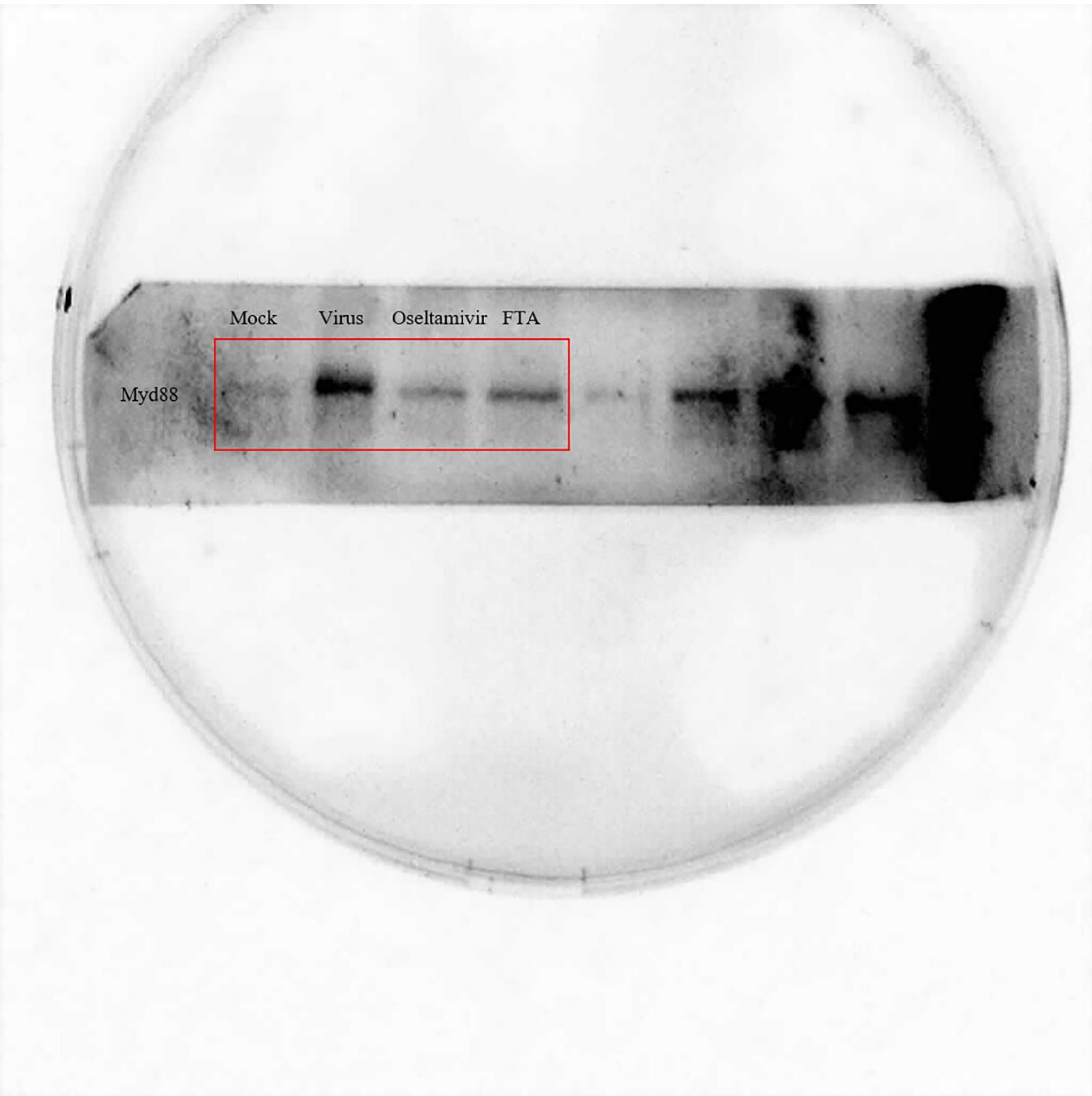

Supplementary Figure3. This figure represents the original picture of Myd88 in the WT mice of Figure 5A in the manuscript.

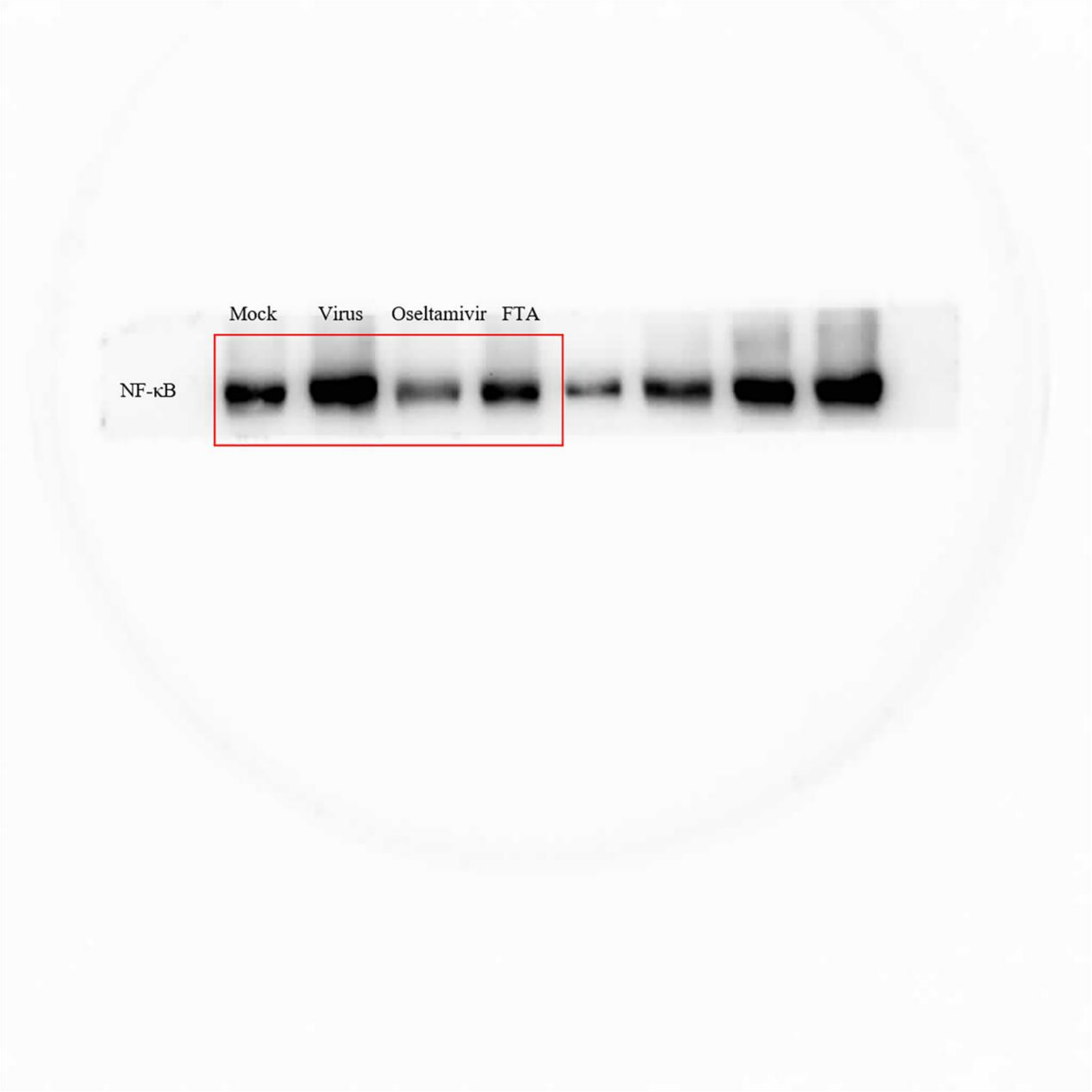

Supplementary Figure4. This figure represents the original picture of NF-κB in the WT mice of Figure 5A in the manuscript.

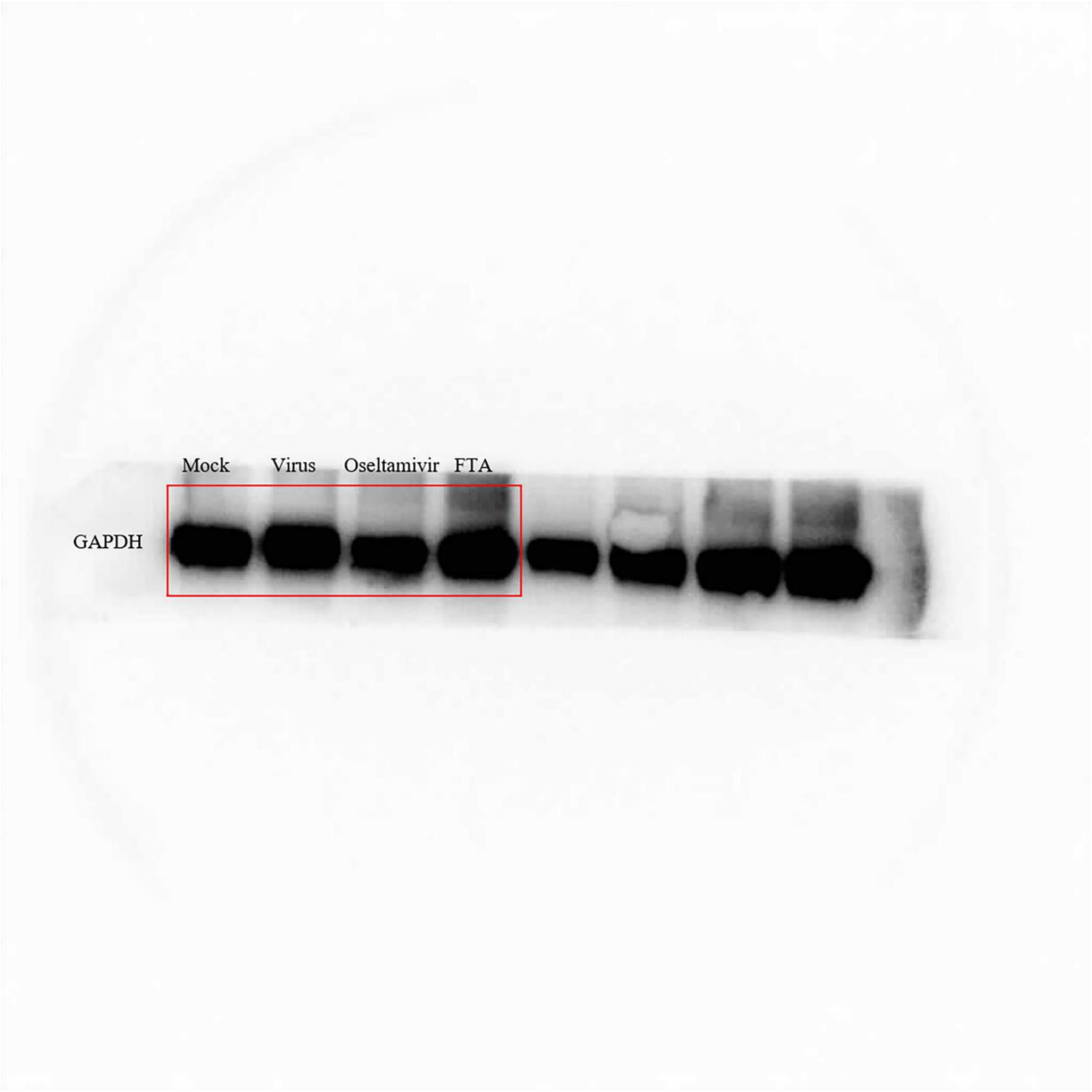

Supplementary Figure5. This figure represents the original picture of GAPDH in the TLR7$^{-/-}$ mice of Figure 5A in the manuscript.

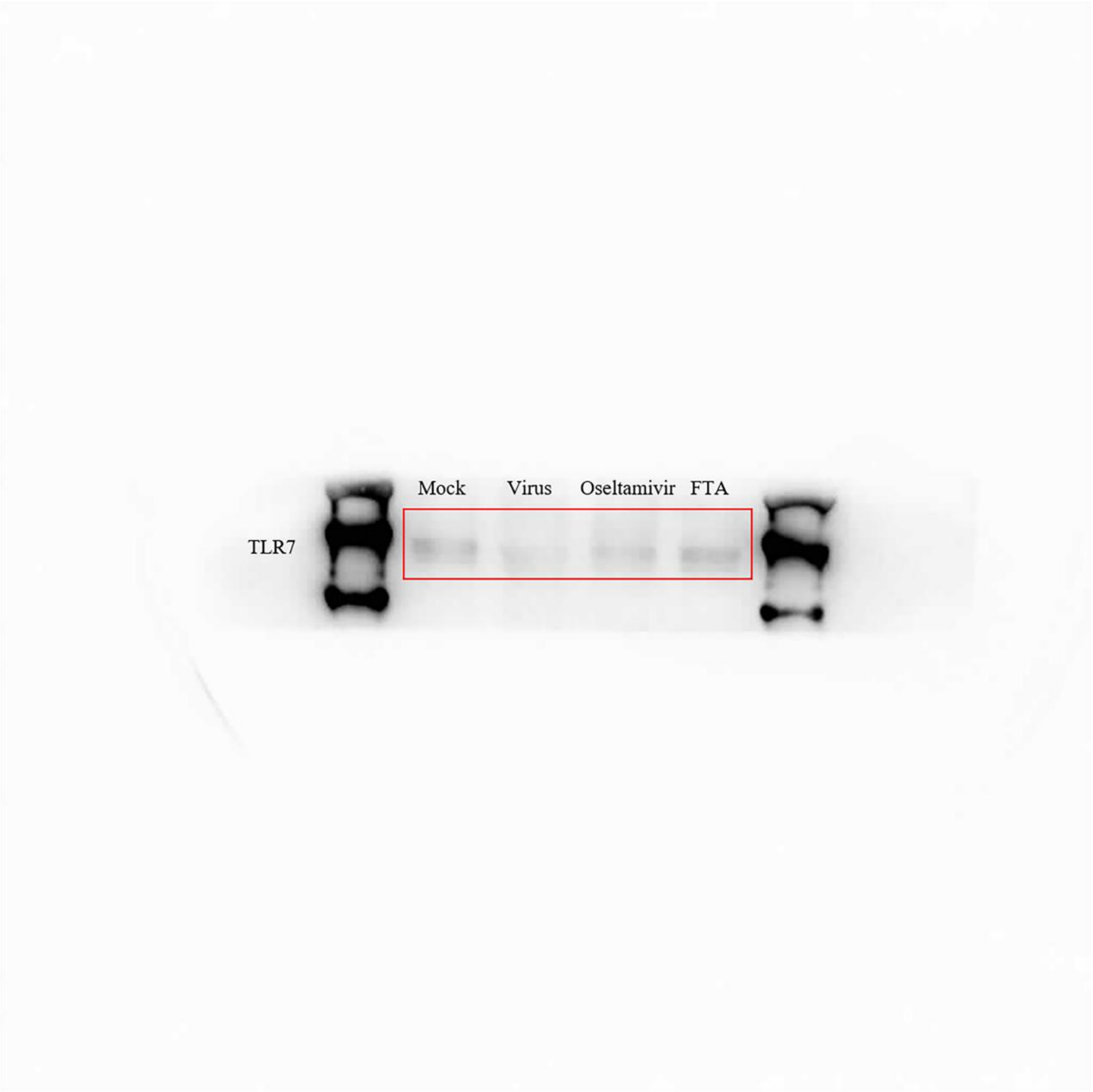

Supplementary Figure6. This figure represents the original picture of TLR7 in the TLR7-/- mice of Figure 5A in the manuscript.

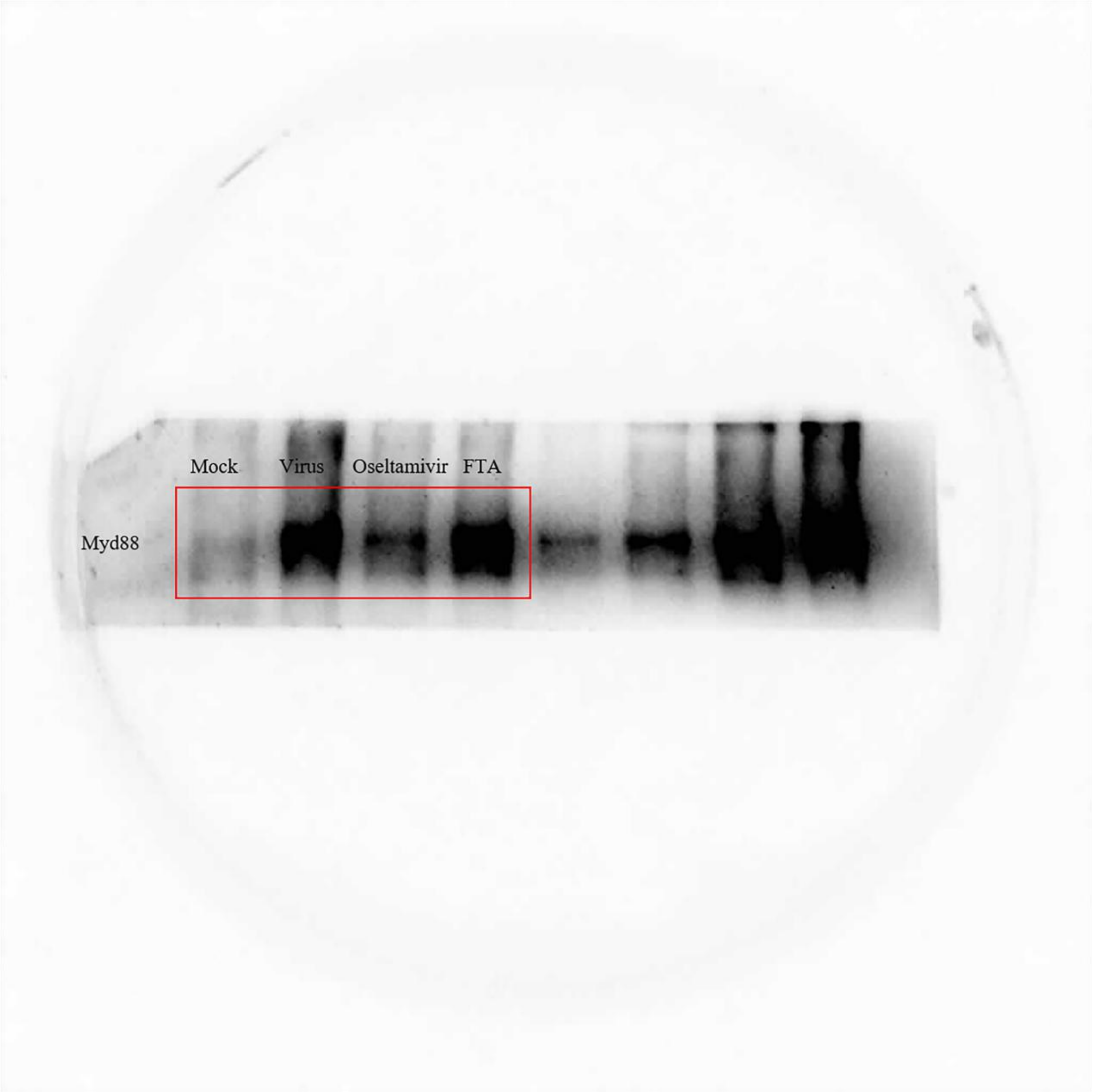

Supplementary Figure7. This figure represents the original picture of Myd88 in the TLR7$^{-/-}$ mice of Figure 5A in the manuscript.

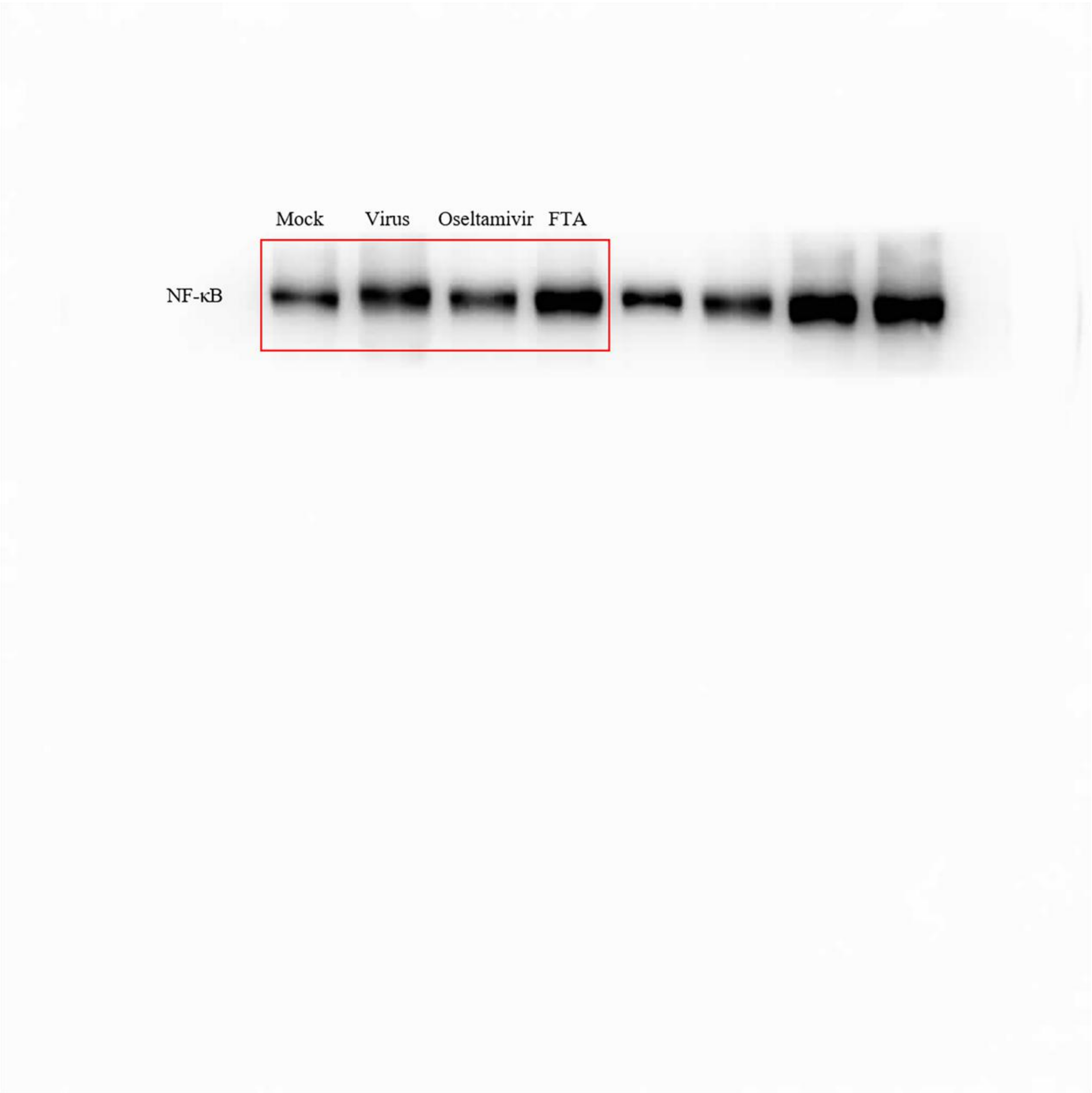

Supplementary Figure8. This figure represents the original picture of NF-κB in the $TLR7^{-/-}$ mice of Figure 5A in the manuscript.
